# Waist Circumference is Associated with Blood Pressure in Children with Normal Body Mass Index: A Cross-Sectional Analysis of 3,417 School Children

**DOI:** 10.5935/abc.20170162

**Published:** 2017-12

**Authors:** Daiane Cristina Pazin, Caroline Filla Rosaneli, Márcia Olandoski, Edna Regina Netto de Oliveira, Cristina Pellegrino Baena, Alyne S Figueredo, Analin Ono Baraniuk, Tatiana Lorena da Luz Kaestner, Luiz Cesar Guarita-Souza, José Rocha Faria-Neto

**Affiliations:** 1 Pontifícia Universidade Católica do Paraná, Curitiba, PR- Brazil; 2 Universidade Estadual de Maringá, Maringá, PR - Brazil

**Keywords:** Child Pediatric Obesity, Waist Circumference, Hypertension, Overweight, Public Health

## Abstract

**Background:**

The prevalence of childhood obesity and associated conditions, such as hypertension, has become a major problem of public health. Although waist circumference (WC) is a marker of cardiovascular risk in adults, it is unclear whether this index is associated with cardiovascular risk factors in children.

**Objective:**

Our aim was to evaluate the association between increased WC and elevated blood pressure (BP) in children with normal body mass index (BMI) ranges.

**Methods:**

Cross-sectional evaluation of students between 6 and 11 years with normal BMI. WC was categorized by quartile for each age group. Normal BP was defined as values < 90th percentile, and levels above this range were considered elevated. Values of p < 0.05 were considered statistically significant.

**Results:**

Of the 5,037 children initially assessed, 404 (8%) were excluded for being underweight and 1,216 (24.1%) were excluded for being overweight or obese. A final sample of 3,417 children was evaluated. The prevalence of elevated BP was 10.7%. In children with WC in the lowest quartile, the prevalence of elevated BP was 8.1%. This prevalence increased in upper quartiles: 10.6% in the second, 12.4% in third and 12.1% in the upper quartile. So, in this group, being in the highest WC quartile was associated with a 57% higher likelihood to present elevated BP when compared to those in the lowest quartile (Q4 vs Q1; OR 1.57 - 95%CI 1.14 - 2.17).

**Conclusion:**

In children aged 6 to 11 years, increased waist circumference is associated with elevated BP even when BMI is normal.

## Introduction

The prevalence of overweight and obesity has increased across all age groups in the last decades, including the pediatric population.^1^ According to the World Health Organization (WHO), more than 40 million children under the age of 5 years were already overweight in 2011. Recent data indicate that almost a quarter of children and adolescents in developed countries are overweight.^2^ Approximately 50% of these overweight children will become overweight adults.^3^ Although the majority of these children live in economically developed countries, the overweight prevalence is also increasing significantly in developing countries.^4^ Together with smoking and hypertension, obesity has become an important cause of preventable deaths worldwide.^5,6^

Genetic and metabolic factors may play a role in the increase of overweight prevalence, which is also directly related to a poor lifestyle, including high calorie intake and sedentary behavior.^7^ The increase in childhood obesity has raised concerns about the development of chronic illnesses that were common in adults and are now emerging in the pediatric population, including early onset of hypertension, glucose intolerance, diabetes, and dyslipidemia, as well as social exclusion and depression.^4,8^ There is also an association of childhood obesity with premature illness and death.^1^ Therefore, the greatest problems of this epidemic, in addition to high costs to health services and great losses to society, will be seen in the next generations of adults.^3,4^

Among the direct consequences of childhood obesity, increased incidence of hypertension is of particular importance.^9^ It predicts premature cardiovascular disease and mortality in adulthood^1,10^ However, abnormal blood pressure (BP) values can also be detected in a percentage of children with normal weight.^11^ Excessive abdominal fat, assessed by waist circumference, has been shown to be an independent risk factor for cardiovascular disease in adults. Though, the association between increased abdominal circumference and elevated BP in children, particularly in normal weight children, has been little explored until recently.^12^

Therefore, our aim in this study was to evaluate the association between increased waist circumference and elevated BP in children between 6 and 11 years of age within normal body mass index (BMI).

## Methods

### Study design and sample

This cross-sectional study was developed using the national registry of children enrolled in public and private schools in the metropolitan region of Maringá, in southern Brazil. This is a city with high human development index (HDI 0.841) and an economy based on agriculture, commerce, and services provision, which is similar to Brazil in general, whose HDI in 2014 was 0.744. The study population included 5,037 school children of both sexes aged between 6 and 11 years. Data was collected by a team of previously trained professionals taking part in the Study and Research Group on Obesity and Exercise from the State University of Maringá (GREPO/UEM), between March and December 2006. The sampling process has been described in a previous publication.^13,14^

The study was approved by the Research Ethics Committee of the State University of Maringá (protocol no. 016/2006) according to the regulations of resolution 196/96 of the National Health Council on scientific research involving human subjects.

### Inclusion and exclusion criteria

The study enrolled children of both sexes with normal BMI, based on reference values for sex and age proposed by Cole et al.^15,16^ Those who refused to participate in data collection or whose parents or tutors did not authorize their participation were excluded. Children absent from school on the day scheduled for data collection and those with inadequate clinical data records were also excluded from the study.

### Data collection

#### Assessment of anthropometric data

##### Assessment of anthropometric data

The children were evaluated for height and weight wearing light clothes (usually the school uniform) and barefoot, without any item that could interfere with the measurements (purse, cap and hair accessories). The mean value of three weight and height measurements was used. Weight and height were measured as described by the WHO^17^ using a Tanita digital scale (2202 model), with a capacity of 136 kg and accurate to 100 g; and a SECA stadiometer (Bodymeter 206 model). Nutritional status was determined based on BMI, according to the sex- and age-specific cut-off values proposed by Cole et al.^15,16^

Waist circumference was measured using an non-elastic metal tape with a precision of 0.1 mm as described by Lohman et al.^18^ It was measured at the end of a normal exhalation with the tape positioned horizontally at the smallest circumference of the torso or midway between the lowest rib and the iliac crest. The measurements were stratified by quartiles to assess the association between circumference and blood pressure. For this purpose, children were initially divided by age group (intervals of 1 year) and then by waist circumference quartile in each age group.

#### BP measurement and definition of elevated BP

BP was measured and categorized according to the guidelines proposed by the Fourth Report on the Diagnosis, Evaluation, and Treatment of High Blood Pressure in Children and Adolescents,^19^ which considers gender, height, and age. BP was measured twice (10-minute interval) using an appropriate cuff, after the child had rested for at least 5 minutes. According to the proposed classification, children are considered normotensive when BP is below 90 percentile, pre-hypertensive (normal-elevated BP) when BP is between the 90^th^ and the 95^th^ percentiles; and hypertensive when BP is equal or above 95^th^ percentile. In the present study, BP values ≥ 90^th^ percentile were defined as "elevated BP".

### Statistical analysis

Data were analyzed using SPSS Statistics for Windows, Version 20.0. Age was described as mean and standard deviation. Qualitative variables were described as frequencies and percentages. The chi-squared test was used to assess the association between waist circumference quartiles and BP (normal or borderline/elevated). One-factor analysis of variance (ANOVA) was used to compare the groups defined by waist circumference quartiles with regard to mean systolic and diastolic blood pressure (SBP and DBP). The correlation between the BMI and waist circumference variables was assessed using Pearson’s coefficient for each age group. Values of p < 0.05 were considered statistically significant.

## Results

### Sample characteristics

Of the 5,037 children initially assessed, 404 (8%) were excluded for being underweight and 1,216 (24.1%) were excluded for being overweight or obese (overweight: 374, 7.4%; obesity: 842, 16.7%), allowing a final sample of 3,417 children with normal BMI. The overall mean age was 8.6 ± 1.3 years and 53,9% were girls. The majority (2,755) was enrolled in public schools. Among the children included in this study, 90.9% reported some physical activity outside of school. Participants’ clinical data and characteristics according to waist circumference quartile are shown in Table 1.

**Table 1:** Characterization of the 3,417 school children with normal body mass indx included in the stud by waist circumference and age

GENERAL			Q1		Q2		Q3		Q4		p
n(%)			Frequency	Percentage	Frequency	Percentage	Frequency	Percentage	Frequency	Percentage	
**Age**											
6 years	402	11.8%	100	11.53%	103	12.19%	99	11.67%	100	16.70%	p = 1
7 years	778	22.8%	204	23.53%	190	22.49%	189	22.29%	195	22.75%	
8 years	791	23.1%	202	23.30%	192	22.72%	196	23.11%	201	23.45%	
9 years	781	22.9%	194	22.38%	197	23.31%	194	22.88%	196	22.87%	
10 years	665	19.5%	167	19.26%	163	19.29%	170	20.05%	165	19.25%	
**Sex**											
Female	1843	53.9%	286	67.01%	460	54.44%	426	50.24%	376	43.27%	p = 0.0000
Male	1574	46.1%	581	32.99%	385	45.76%	422	49.76%	481	56.13%	
**Ethnicity**											
White	3409	99.8%	866	99.88%	842	94.64%	847	99.88%	854	99.65%	p = 0.56499
Non-white	8	0.12%	1	0.12%	3	0.36%	1	0.12%	3	0.35%	
**School**											
Private	662	19.4%	138	15.92%	155	18.34%	168	19.81%	201	23.45%	p = 0.00092
Public	2755	80.6%	729	84.08%	690	81.66%	680	80.19%	656	76.55%	

* The chi-squared test was used for all variables.

### Association between waist circumference and elevated BP

**Figure 1:**
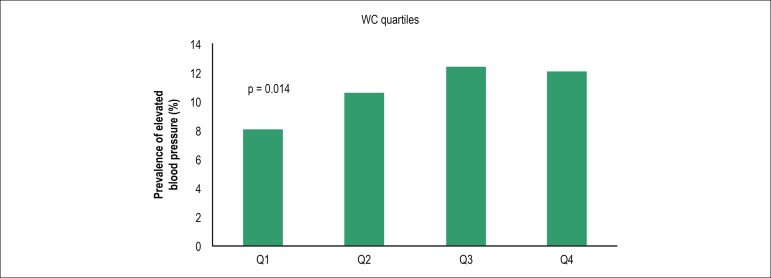
Prevalence of elevated blood pressure according to waist circumference (WC - quartile). Q4 x Q1: OR = 1.57 (95%CI: 1.14 - 2.17) p = 0.014

**Figure 2:**
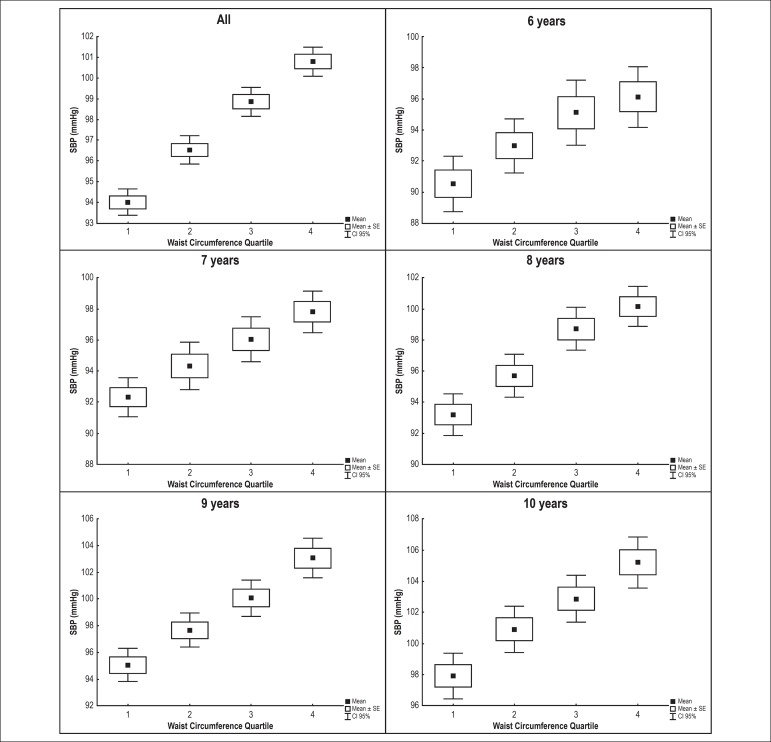
Association between systolic blood pressure (SPB) and waist circumference by age group

**Figure 3:**
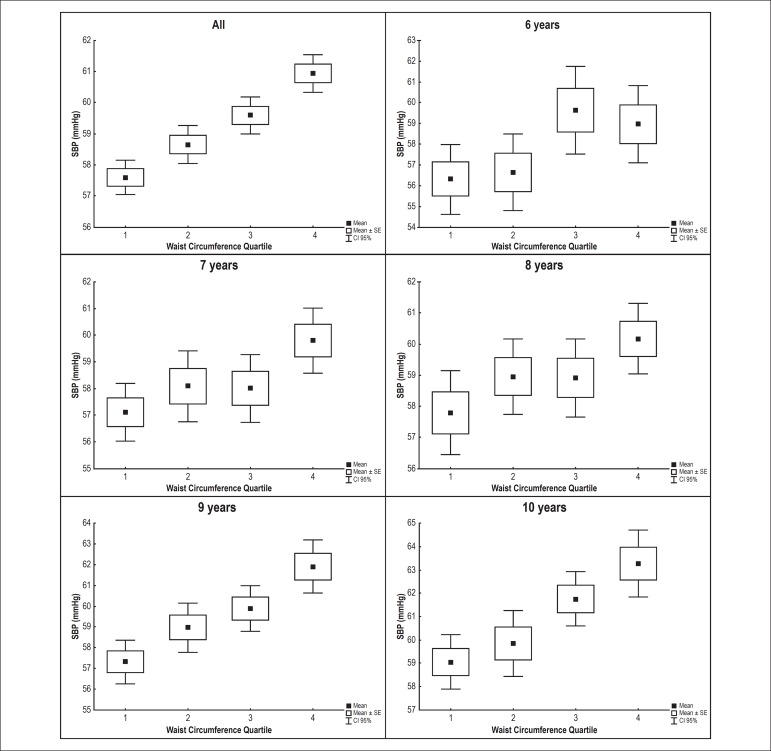
Association between diastolic blood pressure (DBP) and waist circumference by age group

The prevalence of elevated BP in the sample population was 10.7% (n = 368). Children with waist circumferences in the lowest quartile (Q1) for their age range had an 8.1% prevalence of elevated BP. There was a 31% increase in prevalence (10.6%) in the second quartile (Q2). The prevalence increased even further in the highest quartiles, to 12.4% and 12.1% in the third (Q3) and fourth (Q4) quartiles, respectively (p = 0.01) (Figure 1). Therefore, children with normal BMI but waist circumferences in the highest quartile had a 57% increased chance of elevated BP than children with waist circumferences in the lowest quartile (Q4 vs. Q1; OR 1.57; 95% confidence interval [CI] 1.14 - 2.17). Figures 2 and 3 show the correlation between waist circumference and SBP and DBP values for each age group. There was gradual elevation of SBP and DBP with increasing waist circumference for all age groups in these children with normal BMI.

## Discussion

This study demonstrated that increased waist circumference is associated with elevated BP even in children with normal BMI. This association was found in all age groups, even with other factors that could influence the results.

Although secondary forms of hypertension are more common in children than in adults, most cases of mild to moderate hypertension in children do not have an identifiable cause.^20^ The increased incidence of hypertension in the pediatric population in recent decades^2^ is probably directly associated with the increased incidence of obesity.^20,21^ However, the use of BMI as the sole anthropometric measurement to evaluate body fat may not be sufficient to indicate elevated BP.

Increased waist circumference is clearly associated with increased cardiovascular risk in adults. This measurement, easily assessed on clinical examination, is directly associated with increased intraperitoneal fat when measured by imaging methods.^22^ The amount of fatty tissue rather than excess weight itself has been correlated with ill health. The distribution pattern of body fat predicts cardiovascular disease, regardless of the degree of obesity as determined by BMI.^11^ In children, waist circumference may be helpful to identify hypertension,^11,23,24^ changes in the lipid profile, and signs of insulin resistance.^25^ However, the association between increased waist circumference and visceral fat (measured directly using imaging methods) is less clear. Only a few studies correlate waist circumference with imaging methods to assess abdominal fat in the pediatric population. There is a correlation between visceral fat assessed by computed tomography (CT) and BMI^24^ and, according to a study using a small sample,^26^ intra-abdominal fat quantified by CT correlates well with skinfold measurements. Although there is no evidence to suggest a direct association between abdominal fat and waist circumference in children, studies comparing assessment methods indicate that this measurement may be a useful tool for risk assessment in children and adolescents.^25,27,28^

Others have also assessed the association between increased waist circumference and hypertension. In a sample of 1,239 Mexican children between 8 and 10 years of age enrolled in public schools, waist circumference was the main anthropometric measurement associated with hypertension.^26^ Similar results were reported in a sample of Asian children, in which waist circumference was associated with hypertension, independently of BMI.^29,30^

A particular strength of the current study is the large sample size, which allowed assessment of associations after exclusion of children with abnormal BMI. Therefore, the results of this study will provide physicians with important clinical information for the evaluation of children with normal BMI. The division of children according to quartiles within each age group (6 to 7 years, 7 to 8 years, etc.) validates the results for the entire age range. The observed association between BMI and waist circumference in these children suggests that increased waist circumference is not always associated with increased BMI, particularly when the latter is within the normal range.

The design of the current study does not allow the establishment of a causal association between increased waist circumference and elevated BP, but this is a limitation of all cross-sectional studies. An additional limitation in the current study was the absence of an imaging method to assess intra-abdominal fat. However, it was possible to show the importance of waist circumference measurement in children. 

## Conclusion

This study demonstrated that children with increased waist circumference are at increased risk of elevated BP, despite normal BMI. Further studies are necessary to determine the standard values for different age groups in different populations. Also, longitudinal studies are necessary to identify the best tools for early identification of factors related to increased risk of cardiovascular disease in the pediatric population.
